# Development and validation of a food-based diet quality index for New Zealand adolescents

**DOI:** 10.1186/1471-2458-13-562

**Published:** 2013-06-08

**Authors:** Jyh Eiin Wong, Winsome R Parnell, Anna S Howe, Katherine E Black, Paula ML Skidmore

**Affiliations:** 1 Department of Human Nutrition, University of Otago, Dunedin 9054, New Zealand; 2 Nutritional Sciences Programme, School of Healthcare Sciences, Faculty of Health Sciences, Universiti Kebangsaan Malaysia, Kuala Lumpur 50300, Malaysia

**Keywords:** Diet quality index, Dietary patterns, Validity, Adolescents, New Zealand

## Abstract

**Background:**

As there is no population-specific, simple food-based diet index suitable for examination of diet quality in New Zealand (NZ) adolescents, there is a need to develop such a tool. Therefore, this study aimed to develop an adolescent-specific diet quality index based on dietary information sourced from a Food Questionnaire (FQ) and examine its validity relative to a four-day estimated food record (4DFR) obtained from a group of adolescents aged 14 to 18 years.

**Methods:**

A diet quality index for NZ adolescents (NZDQI-A) was developed based on ‘Adequacy’ and ‘Variety’ of five food groups reflecting the New Zealand Food and Nutrition Guidelines for Healthy Adolescents. The NZDQI-A was scored from zero to 100, with a higher score reflecting a better diet quality. Forty-one adolescents (16 males, 25 females, aged 14–18 years) each completed the FQ and a 4DFR. The test-retest reliability of the FQ-derived NZDQI-A scores over a two-week period and the relative validity of the scores compared to the 4DFR were estimated using Pearson’s correlations. Construct validity was examined by comparing NZDQI-A scores against nutrient intakes obtained from the 4DFR.

**Results:**

The NZDQI-A derived from the FQ showed good reliability (r = 0.65) and reasonable agreement with 4DFR in ranking participants by scores (r = 0.39). More than half of the participants were classified into the same thirds of scores while 10% were misclassified into the opposite thirds by the two methods. Higher NZDQI-A scores were also associated with lower total fat and saturated fat intakes and higher iron intakes.

**Conclusions:**

Higher NZDQI-A scores were associated with more desirable fat and iron intakes. The scores derived from either FQ or 4DFR were comparable and reproducible when repeated within two weeks. The NZDQI-A is relatively valid and reliable in ranking diet quality in adolescents at a group level even in a small sample size. Further studies are required to test the predictive validity of this food-based diet index in larger samples.

## Background

Research into diet-disease relationships has recently shifted from a traditional focus on the role of single nutrients or foods groups to the emphasis on dietary patterns or diet quality [1,2]. Dietary pattern analysis facilitates the examination of multiple dietary components including nutrients, foods and food groups as a combined exposure, and is therefore a holistic alternative to the single nutrient or food approach [3,4]. Using an *a-priori* approach, a diet index provides a summary of dietary patterns as a composite score according to predefined criteria of what constitutes a healthy or unhealthy diet [5,6]. Based on foods, nutrients or a combination of both, a diet index typically measures the degree of adherence to a set of national nutrition guidelines or a recommended diet prototype such as the Mediterranean diet [7]. As better index scores have been positively associated with more favourable nutrient and food intakes and lower all-cause mortality and disease risks [4,6], most diet indices have been used as indicators of overall diet quality.

In New Zealand (NZ), adolescents aged 15 to 18 years have been shown to have less healthy diets compared to older age groups [8]. Findings from the recent 2008/09 NZ Adult Nutrition Survey revealed a high proportion of adolescents with nutrient intakes that did not meet the appropriate Nutrient Reference Values [8]. In addition, their diets were characterised by lower servings of vegetables and wholegrain breads than are recommended and highest frequency of consumption of hot chips and sugar-sweetened drinks among all age groups [8]. While information on nutrients and food group sources are presented [8,9], no assessment has been made of the overall diet quality of NZ adolescents using diet quality indices. International data on diet quality for this age group is also scarce [10].

The assessment of diet quality among adolescents has tended to use both food and nutrient-based diet indices such as the Healthy Eating Index [11-13] and its derivatives [14,15]. These diet indices were adapted from those originally developed for adults based on the United States Dietary Guidelines. Other frequently used diet indices evaluate diet quality by the level of adherence to the principles of Mediterranean dietary patterns [16-18] and regional or country-specific dietary guidelines [19,20]. While the nutrient recommendations for NZ are similar to, or the same as other countries like Australia [21], the food-based dietary guidelines are different. Existing diet indices neither reflect the typical foods consumed by the NZ population nor the country’s food guidelines, therefore they are not suitable for direct application in NZ. Given the differences in food choices and nutrient intakes between NZ adolescents and adults [8], it is probable that a diet index based on adult population data would not be applicable to adolescents.

In addition, most work on diet indices in adolescents has been limited to large surveillance and longitudinal studies where detailed and quantitative measures of food intakes were available for derivation of diet index scores [22-24]. With the increasing use of short dietary assessment methods in adolescent populations [25], there is a need for simple, easy-to-apply diet indices based on these methods of dietary assessment that address country-specific dietary guidelines to allow assessment of diet quality at population level. However, few studies have derived diet quality indices using non-quantitative food frequency questionnaires (FFQs) [26,27] and brief questionnaires [28]. Any new dietary assessment tool should be validated against an independent reference method [29]. Similarly, a newly developed diet index should be validated by comparing the scores obtained from the tool used to create the index against scores calculated from a more in-depth dietary assessment method with uncorrelated errors [30]. However, the majority of published diet indices have been validated against the same dietary assessment method used to create the index [30]. This potentially leads to an over-estimation of validity. Moreover, the reliability of a diet index has seldom been reported [31].

Therefore, the aims of this study were to develop an adolescent-specific, food-based diet quality index (New Zealand Diet Quality Index for Adolescents, NZDQI-A) based on dietary information obtained from a brief food questionnaire and to assess the test-retest reliability and relative validity of the NZDQI-A.

## Methods

### Study design and participants

A convenience sample of adolescents aged 14 to 18 years volunteered to participate in this validation study. Participants were recruited via schools, sports clubs and youth groups in Dunedin, NZ between November 2010 and May 2011. The study protocol was reviewed and approved by the Human Ethics Committee of the University of Otago (Reference 10/131). All participants provided written informed consent and parents had the opportunity to decline their children’s participation by signing an ‘opt-out’ consent form prior to their involvement in the study.

### Dietary assessment tools

#### ***Food questionnaire***

Habitual food intake was estimated using a Food Questionnaire (FQ) which was repeated within two weeks. The FQ comprised two sections: summary questions and the NZ Adolescent Food Frequency Questionnaire (NZAFFQ) [32]. In the first section, participants were asked five summary questions on the number of daily servings they consumed from the following four food groups: ‘fruits’, ‘vegetables’, ‘breads and cereals’, and ‘meat and alternatives’. One serving of a food group was defined using the definitions specified in the New Zealand Food and Nutrition Guidelines (NZFNG) for Healthy Adolescents [33]. Examples of a standard serving size were provided for each question. The response categories were ‘none’, ‘less than one per day’, ‘1 serving’, ‘2 servings’, ‘3 servings’ and ‘4 or more servings’. For breads, the frequency responses ranged from ‘none’ to ‘7 or more per day’. Items such as pasta, rice, muesli, porridge and breakfast cereals were considered as cereals, and servings per week was asked. These summary questions have previously been used in National Nutrition Surveys within NZ [8,34]. In section two (NZAFFQ), participants indicated their usual intakes of 72 food items in the past four weeks by selecting one of the seven frequency categories ranging from ‘none’ to ‘more than once per day’. For fruits, vegetables, and snack foods, consumption in the past seven days was obtained. These frequency questions have been shown to be valid and reliable when completed twice over a two-week period in estimating food group intakes in this adolescent sample [32].

#### ***Four-day estimated food record***

Each participant was asked to keep a four-day estimated food record (4DFR) as a reference method for comparison with the first FQ. Participants were given written instructions with the food records and were taught how to record intakes of foods and beverages for three weekdays and one weekend day. To facilitate estimates of portion size, participants were each given a measuring cup, centimetre ruler, circle diameter and food portion size photographs, as used in previous National Nutrition Surveys [35].

The foods recorded in the 4DFR were coded to the corresponding food groups from the NZAFFQ for calculation of the 4DFR based NZDQI-A (Additional file 1). For mixed dishes, proportions of component ingredients were calculated and assigned to one of the 26 food sub groups. To ensure consistency in data-entry decisions, 4DFRs were entered by one trained nutritionist and counter-checked by another. Food intakes were converted to weights (g) before being entered into the NZ dietary analysis software Diet Cruncher (Way Down South Software, Dunedin, NZ) for nutrient calculation based on the 2006 New Zealand Food Composition database (FOODfiles) [36]. All food data from the 4DFR were entered for calculation of nutrients.

#### ***Development of the New Zealand Diet Quality Index for Adolescents***

##### 

**Figure 1:**
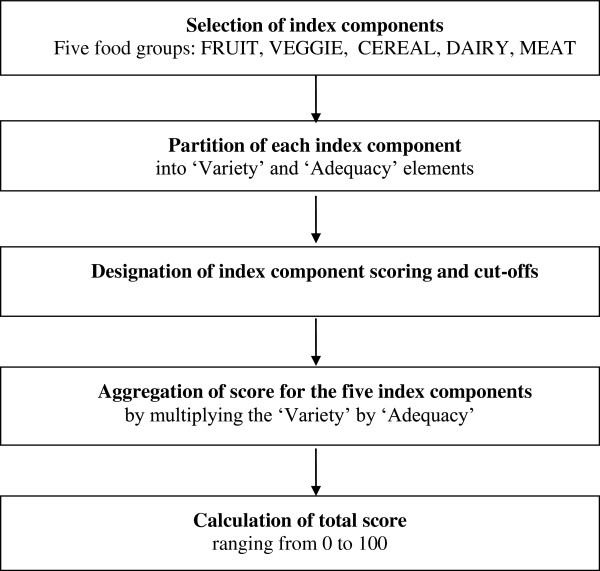
Development of the New Zealand Diet Quality Index for Adolescents (NZDQI-A).

**Index components selection** When selecting a suitable index component, two main aspects were considered (Figure 1). First, all dietary information that makes up the index components needed to be available within the FQ. As the NZAFFQ (section Methods of the FQ) does not contain portion size information, quantification of nutrient intakes was not possible, so the computation of scores was based solely on summary questions and food frequencies. Secondly, the index components needed to be based on the NZFNG for Healthy Adolescents [33], and should provide a reflection of overall diet quality.

Considering these aspects, the food-based NZDQI-A comprised five index components, each representing a major food group: fruits (FRUIT), vegetables (VEGGIE), bread and cereals (CEREAL), milk and milk products (DAIRY), and meat and alternatives (MEAT). As the NZDQI-A employed a five-food-group concept, only data from the five summary questions and 53 food frequency questions relating to these food groups from the FQ were included for scoring of the NZDQI-A (see Additional file 1).

The NZDQI-A was constructed to represent two key elements of diet quality: (1) diet variety (‘Variety’), and (2) diet adequacy (‘Adequacy’). Diet variety is universally recognized as an important principle underlying a healthy diet [37]. It has been recommended as part of many national dietary guidelines including the NZFNG due to its role in increasing exposure to a wide range of nutrients thereby enhancing nutrient adequacy [38]. As indicated by the first NZFNG statement ‘Eat many different kinds of food each day’ [33] variety both within and across food groups is considered important. There is a lack of uniformity in the definitions and methods used to define diet variety in previous research [38,39]. ‘Variety’ was therefore broadly defined as the number of different food sub groups consumed over a week (see Additional file 1).

In this study, ‘Adequacy’ reflects adherence to the serving recommendations for all major food groups (fruits and vegetables, bread and cereals, milk and milk products and meat and alternatives) outlined in the NZFNG for Healthy Adolescents [33]. Consuming the recommended quantity in terms of numbers of servings from each of the food groups should mean that adolescents will meet nutrient recommendations [40], which may in turn lower the risk of nutrient deficiencies [21]. This element of diet quality has been incorporated in many published diet quality indices [41-43].

#### ***Scoring and cut-offs***

The scoring criteria for the NZDQI-A are described in Table 1. For each index component, a score was calculated by multiplying the ‘Variety’ and ‘Adequacy’ elements. ‘Variety’ was calculated as the proportion of food sub groups consumed from within a particular food group in a week, regardless of portion size [44]. To be counted as contributing variety, an intake of at least ‘once a week’ in the NZAFFQ was specified. With regard to ‘Adequacy’, the adolescent-specific recommended daily servings for each food group [33] were used to create the cut-off range. For FRUIT and VEGGIE components, the recommended daily servings were at least two and three servings per day, respectively. For the CEREAL component, at least six daily servings of all breads, cereals, rice and pasta were recommended. The cut-offs were set at three servings per day for the DAIRY component and one to two servings per day for the MEAT component. Previous research showed that employment of summary questions to be a valid approach in deriving food-based diet indices [19,42], therefore ‘Adequacy’ was scored for four index components based on responses to the five summary questions in the FQ. As there is no such summary question available for milk and milk products due to difficulty in standardising serving size for various dairy foods, frequency questions were used to estimate the intake of the dairy servings for the DAIRY component.

**Table 1:** Components and scoring of the New Zealand Diet Quality Index for Adolescents (NZDQI-A)

**Component**	**Elements of NZDQI-A**				**Criteria to achieve maximum component score**^**4**^
	**‘Variety’**	**‘Adequacy’**			
	**Score**^**1 **^**(v/V)**	**Indicators in the FQ**^**2**^	**Cut-offs**^**3**^	**Score (A)**	
FRUIT	v/6	Servings of fruit per day^5^.	0 serving/day	0	Consumed at least 2 daily servings of fruits from 6 varieties in a week.
			< 2 serving/day	10	
			≥ 2 servings/day	20	
VEGGIE	v/6	Servings of vegetables per day^6^.	0 serving/day	0	Consumed at least 3 daily servings of vegetables from 6 varieties in a week.
			< 3 servings/day	10	
			≥ 3 servings/day	20	
CEREAL	v/3	Servings of bread per day.	0 serving/day	0	Consumed at least 6 daily servings of cereals from 3 varieties in a week.
		Servings of pasta, rice, muesli, porridge or breakfast cereals per week.	< 6 servings/day	10	
			≥ 6 servings/day	20	
DAIRY	v/4	Frequency intake of milk (standard and non-standard milk), flavoured milky drink, cheese and yoghurt^7^	0 serving/day	0	Consumed at least 3 daily servings of milk or milk products from 4 varieties in a week.
			< 3 servings /day	10	
			≥ 3 servings/day	20	
MEAT	v/7	Servings of meat, chicken, seafood, eggs or meat alternatives eaten per day.	0 serving/day	0	Consumed 1 or 2 daily servings of meat or alternatives (not including processed meats) from 7 varieties in a week.
			< 1 serving/day	5	
			1-2 servings/day	20	
			> 2 servings/day	10	
Total Score	= Σ (v/V) x A = FRUIT + VEGGIE + CEREAL + DAIRY + MEAT				

Food Questionnaire (FQ), Fruits (FRUIT), Vegetables (VEGGIE), Bread and cereals (CEREAL), Milk and milk products (DAIRY), Meat and alternatives (MEAT).

^1^ Ratio calculated as the different sub groups (v) consumed at least once in a week (as indicated in the NZAFFQ) divided by the total sub groups (V) in a food group. The food sub groups are outlined in Additional file 1.

^2^ Refers to questions in the FQ.

^3^ Based on achievement of the recommended daily servings as suggested by the Ministry of Health [33].

^4^ For each component, a total score is calculated by multiplying ‘Variety’ (v/V) by ‘Adequacy’ (A). The possible score range is 0 to 20. E.g. For a person who consumes at least two daily servings of fruits from three varieties in a week, FRUIT score = (3/6) × 20 = 10.

^5^ Include fresh, frozen, canned and stewed fruits.

^6^ Include fresh, frozen and canned vegetables, including potatoes.

^7^ Weekly frequency of intake (times per week) for the four sub groups as reported in the NZAFFQ. Weekly frequencies were summed and converted into daily frequencies. One time per day was equivalent to one serving.

For all components, a maximum ‘Adequacy’ score of 20 was assigned to participants who met the guidelines for each of the five food groups while non-consumers were scored zero. Except for the MEAT component, participants who consumed less than the recommended servings of a particular food group were scored 10 for ‘Adequacy’. Due to the observed U-shaped association between meat and health [5], moderate consumption of meat was deemed beneficial. Thus for the MEAT component, a subtraction approach was used where by consumption of one to two servings of meat or alternatives in a day were given a score of 20 and consumption beyond this range was given a score of 10. Each individual index component was scored from 0 to 20, with a higher score reflecting greater adherence to dietary recommendations in terms of variety and intake servings for a food group. The total score was the sum of the five equally weighted index components converted to a possible score between 0 and 100.

#### ***Data and statistical analysis***

Using the Shapiro-Wilk and skewness tests, the normality of energy and nutrient variables from the 4DFR were determined. The 16 selected nutrients were protein, total fat, saturated fat (SFA), monounsaturated fat (MUFA), polyunsaturated fat (PUFA), carbohydrate, total sugars, glucose, lactose, fructose, maltose, sucrose, dietary fibre, calcium, iron and vitamin C. These variables were presented as means or geometric means if log-transformed. Macronutrient intakes were adjusted for total energy intake (MJ) using the residual method [45] to ensure that nutrient intakes were independent of energy intakes. Cronbach’s alpha statistic was used to examine how well the individual index components fit together in measuring the same construct. Conventionally, an alpha coefficient above 0.70 is considered desirable [46], however lower values are acceptable for indices formed by less than 10 components [47]. An overall Cronbach’s alpha coefficient of 0.5 or higher was deemed sufficient to indicate that the index components have adequate internal reliability in measuring diet quality.

#### ***Relative validity and test-retest reliability***

Correlations between the NZDQI-A scores derived from the first FQ and those derived from the 4DFR were examined using Pearson’s correlation coefficients. Based on expert recommendations and results of previous research, acceptable correlation coefficients for diet indices used in adolescents range broadly from 0.20 to 0.66 [28,48-51]. Following grouping of participants into thirds of total NZDQI-A scores, cross-classification analysis was used to measure the agreement between the two methods in ranking participants. The percentage of participants correctly classified into the same thirds and misclassified into extreme thirds was identified. For test-retest reliability, Pearson’s correlations and cross-classification analysis were computed using the scores derived from the first and second FQs. For all non-normally distributed component scores, the non-parametric equivalent (Spearman’s correlations) was used.

#### ***Construct validity***

We hypothesised that there would be a positive relationship between higher NZDQI-A scores calculated from the FQ and more favourable nutrient intakes reported by the 4DFRs. Non-parametric analysis of linear trend across ordered groups (*nptrend* command in Stata) was employed to examine the ability of the NZDQI-A to rank participants by nutrient intakes across the thirds of score. All analyses were performed using the statistical program Stata version 11.2 (2009, Stata Corporation, Texas). P values of less than 0.05 were considered significant.

## Results

### Participant characteristics

Of the initial 79 people who agreed to take part in this study, 38 completed all parts of the study (4DFR and repeated FQs), 3 completed a 4DFR and a single FQ, and 14 completed only the two FQs. In total, 41 participants (16 males, 25 females) were included in the validity (relative and construct validity) analysis while 52 participants (28 male, 24 female) were included in the test-retest reliability analysis. The mean age of participants was 15.0 ± 0.8 years and ranged from 14.0 to 17.9 years. The mean NZDQI-A score was 52.5, ranged from 22.0 to 84.2. Male participants achieved a mean NZDQI-A score of 55.7 (SD 15.6) and the mean score was 50.4 (SD 12.1) for female participants. There was no significant difference between males and females in the NZDQI-A total and component scores (p > 0.05).

### New Zealand Diet Quality Index for Adolescents

The Cronbach’s alpha coefficient for the total score was 0.51, which indicated that the NZDQI-A had a fair internal consistency in measuring diet quality. This alpha coefficient was considered acceptable due to the complex multidimensional nature of diet quality [52]. Participants who achieved high total NZDQI-A scores may not necessarily had scored high consistently for all the five food group components. As noted in Methods section, an alpha coefficient lower than 0.7 was not unexpected as the NZDQI-A comprised less than ten index components.

### Relative validity and test-retest reliability

The validity of the NZDQI-A scores derived from the FQ relative to those from the 4DFR are presented in Table 2. Correlation coefficients showed that both methods had fair agreement in ranking the NZDQI-A total score (r = 0.39). The correlation coefficients for individual components ranged from 0.21 to 0.57. In general, more than half of the participants were classified into the same thirds while 10% were misclassified into the opposite thirds. The NZDQI-A total score derived from the repeated FQs showed good reproducibility (r = 0.65), with reliability coefficients ranging from 0.32 to 0.67 for the individual components. Test-retest reliability was highest for FRUIT, but lowest for the MEAT component.

**Table 2:** Relative validity and test-retest reliability of the New Zealand Diet Quality Index for Adolescents

	**Relative validity (n = 41)**			**Reliability (n = 52)**		
**Component**	**CC**^**1**^	**% CC**	**% GM**	**CC**^**2**^	**% CC**	**% GM**
FRUIT	0.28	39	15	0.67	62	4
VEGGIE	0.21	34	5	0.58	62	2
CEREAL	0.57	63	2	0.56	90	10
DAIRY	0.40	39	15	0.63	73	10
MEAT	0.27	27	7	0.32	44	12
Total Score	0.39	51	10	0.65	60	6

Correlation coefficients (CC), percent correctly classified (% CC), percent grossly misclassified (% GM), Fruits (FRUIT), Vegetables (VEGGIE), Bread and cereals (CEREAL), Milk and milk products (DAIRY), Meat and alternatives (MEAT).

^1^ Pearson’s correlations for FRUIT, MEAT and Total Score while Spearman’s correlations for VEGGIE, CEREAL and DAIRY between FQ and 4DFR.

^2^ Pearson’s correlations for FRUIT, MEAT and Total Score while Spearman’s correlations for VEGGIE, CEREAL and DAIRY between first and second FQs.

### Construct validity: association between scores and nutrient intakes

When comparing nutrient intakes across the thirds of NZDQI-A score, those in the top third for NZDQI-A scores had higher intakes of iron and lower intakes of total fat, SFA and MUFA. Higher total scores were also associated with higher total sugars and fructose in the trend analysis (Table 3).

**Table 3:** Nutrient intakes according to distribution thirds of the New Zealand Diet Quality Index for Adolescents (n = 41)

**Energy and nutrients**^**1**^	**Thirds of NZDQI-A**			**P for trend**^**2**^
	**Low**	**Medium**	**High**	
	**(22.0 - 49.4)**	**(49.9 - 57.4)**	**(57.6 - 84.2)**	
Energy (kJ)	7542	7216	8171	NS
Protein (g)	67.5	74.8	75.7	NS
Protein (% energy)	15.2	17.1	17.1	NS
Total fat (g)	70.4	62.4	59.0	<0.01
Total fat (% energy)	34.5	30.1	29.6	<0.05
SFA:PUFA ratio	3.89	3.52	2.85	<0.05
SFA (g)	30.5	26.3	23.4	<0.01
MUFA (g)	23.6	20.6	19.9	<0.01
PUFA (g)	8.2	7.9	8.6	NS
Cholesterol (mg)	197	181	243	NS
Carbohydrate (g)	228.4	240.2	244.1	NS
Carbohydrate (% energy)	48.2	50.8	51.3	NS
Total sugars (g)	98.3	113.7	119.7	<0.05
Glucose (g)	15.1	18.0	19.3	0.050
Lactose (g)	9.4	12.3	9.0	NS
Fructose (g)	18.1	19.1	22.9	<0.05
Maltose (g)	2.9	3.2	3.6	NS
Sucrose (g)	48.0	56.5	58.5	NS
Dietary fibre (g)	17.4	18.9	17.8	NS
Calcium (mg)	583	702	681	NS
Iron (mg)	9.6	10.2	13.3	<0.05
Vitamin C (mg)	82.5	82.5	92.7	NS

New Zealand Diet Quality Index for Adolescents (NZDQI-A), saturated fat (SFA), monounsaturated fat (MUFA), polyunsaturated fat (PUFA), not significant (NS).

^1^ All nutrients except for calcium, iron and vitamin C were adjusted for total energy intake using the residual method [45]. Values are presented as means or geometric means if log-transformed.

^2^ P value for trend across the thirds using the non-parametric trend analysis.

## Discussion

This is the first study in NZ that has used a diet index to assess diet quality in adolescents, and one of the first to have simultaneously addressed the relative and construct validity of an FQ-derived DQI by utilising different dietary assessment methods. Unlike the majority of studies which have used the same instrument for constructing and validating a diet index, the present study has the advantage of having examined the validity of the NZDQI-A using an independent measure of nutrient intakes, i.e. 4DFR data.

### Relative validity and test-retest reliability

The results of this study showed that Pearson’s correlation coefficient for the FQ-derived NZDQI-A score relative to those from the 4DFR (r = 0.39) fell within the range of 0.20 to 0.66 as observed in previous validation studies [28,48-51]. At least 50% of participants were correctly classified into the exact thirds by the two methods. Among the five index components, the relative validity was highest for CEREAL, but lowest for VEGGIE. Poor agreement for individual index components may be attributed to the small sample size or assignment of a narrow score range (0 to 20), which may have grouped participants with very similar scores into different thirds [53]. Nevertheless, as diet quality is determined by the collective contribution of the five food groups, the relative validity of individual components may be less important than the relative validity of the total NZDQI-A score. This may also imply that comparison between two methods should only be made using the total score. For reliability, the correlation coefficients were above 0.5 for the total and component scores apart from the MEAT component, suggesting acceptable test-retest reliability of the NZDQI-A scores when repeated over a two-week period.

### Construct validity

To establish the construct validity of the index as an indicator of diet quality, we compared the NZDQI-A scores to nutrient intakes derived from the 4DFRs (Table 3). Significant trends towards better diet quality were observed with increasing NZDQI-A scores, as evident by the increased intake of iron but decreased intake of fat (total and percent of energy) across the thirds of scores. It is also important to note that the observed trend in fat intake was independent of energy intake, suggesting that the amount of food consumed did not influence the diet quality score.

Increasing NZDQI-A score was associated with lower total fat and SFA intakes. Although the decline in total fat intake was also parallel to lower MUFA intake, our analysis suggested that the decline was more likely to be indicative of an increased fat quality, as shown by the significant decreasing trend of saturated-to-polyunsaturated fat ratio over the thirds of the NZDQI-A (p = 0.02). For total sugars, the increase was likely to be influenced by the higher fruit intakes, as indicated by higher fructose levels across the thirds of scores. This observation is in accordance with results of a recent national nutrition survey which showed that fruits and non-alcoholic beverages (including fruit juices) were the main sugar sources for NZ adolescents aged 15 to 18 years [8].

### Comparisons with other studies

Although an increasing number of studies have employed diet indices to describe diet quality in children and adolescents [12-14,16-20,22-24,28,51,54-56], none have been conducted in NZ. Six studies have reported the validity of a diet index against an independent reference method among populations which included adolescents aged 13 years and above [17,20,23,28,51,54]. Among these six studies, three studies validated their indices using nutritional biomarkers [20,23,54] including the large-scale European HELENA study [20]; whilst the other three studies validated against diet records [51] and 24-hour diet recalls [17,28].

The first study by Torheim *et al.*[51] compared the validity of the Food Variety Score and Diet Diversity Score calculated from two FFQs (69 and 164 food items) to those from 2-day weighed records. Correlation coefficients between the two methods ranged from 0.2 to 0.5 for the two indices of diet variety. In another study by Schroder *et al.*[28], dietary intakes of 11 selected nutrients reported by multiple 24-hour recalls were found to be positively associated with the three diet quality indices (Diet Quality Index, modified Mediterranean Diet Score and Antioxidant Score) derived from two short questionnaires containing 15 to 18 food items. The validity correlation coefficients between the two methods ranged from 0.32 to 0.45. In these two studies, validation work was completed in relatively large samples with wide age ranges (n = 145 aged 15 to 59 years and n = 102 aged 3 to 80 years, respectively). As results were not reported separately for adolescents, a direct comparison with these studies was not possible. In spite of this, we found that our NZDQI-A calculated from a FQ produced similar if not better correlations plus reasonable agreement in ranking scores compared to a 4DFR. In the validation study by Serra-Majem *et al.*[17] among 3166 children and adolescents (aged 6 to 24 years), an increased mean intake for a majority of vitamins and minerals calculated from 24-hour diet recalls was found with increasing Mediterrannean diet adherence score using the KIDMED. Employing a similar analytical approach in our study, the significant trends towards more optimal intakes of selected nutrients are also suggestive of the NZDQI-A’s construct validity.

Following expert recommendations [5,7], the current NZDQI-A was constructed based on five major food groups to reflect the fundamental premise of the NZ food-based dietary guidelines, which emphasised that nutrient needs should be met primarily through ‘eating different kind of foods each day’ [33]. To address the equal importance of having variety in addition to adequacy in diets, a total score was calculated by multiplying both construct elements for the five equally weighted index components.

The methodology used for constructing the NZDQI-A may have implications for validity estimates in this study. First, as this index was constructed within the boundaries of the FQ data, the limited number of questions in the questionnaire may have restricted the variation in food variety and serving intake responses. In particular, our NZAFFQ data does not distinguish between wholegrain and refined grains and between lean and fatty meats; hence the index scoring was not specific to carbohydrate and fat quality. In any case, participants who scored higher in this study seemed to consume less fat and better fat quality, but no relationship was seen for dietary fibre. To improve the ability of the NZDQI-A to detect fibre intake, a possible index modification may include a component that gives merit to a higher intake of wholegrain bread relative to white bread, given that breads are reported to be the main dietary fibre source for NZ adolescents [8].

Secondly, the current NZDQI-A components were selected to reflect positive food choices rather than negative ones. Therefore in measuring favourable food group intakes, the index discounts excess intake of foods high in fat, sugar and salt. This may then mean that it is possible that a higher quality index score is reflective of a higher energy intake. However, results from this study suggest that this is not the case. Some authors have used a deduction approach to demerit foods considered detrimental such as sausages, pastries, confectionery, soft drinks and fast foods in their composite index [23,28]. Nevertheless, unless population-specific evidence-based recommendations for these discretionary foods are available, the decision on what is considered ‘an acceptable intake level’ of such foods remains subjective.

With regard to scoring of the index, we used trichotomous cut-off points (0 for non-consumption, 10 for intakes below recommendations and 20 for intakes in line with recommendations) based on the suggested minimum servings of the dietary guidelines [33]. We are conscious that this scoring approach has disadvantages. Mainly, the discriminating power of the NZDQI-A may potentially be reduced when most participants have low intakes of certain food groups [57], especially when the sample size is small. For instance, only 22% of participants achieved the suggested daily intake of milk and dairy products in this study. The low intakes of this food group resulted in more than 80% of participants scoring 10 and below for the DAIRY component. The right-skewed distribution of this component score may have diluted the resultant total score and therefore attenuated the association between the NZDQI-A and calcium intakes in the sample.

Lastly, the validity of a diet index is reflective of the dietary guidelines upon which it is based [50,58]. When possible, quantitative criteria were used in establishing the cut-off points for scoring the NZDQI-A. This was however not feasible for all foods, as some intake recommendations were not quantified in the NZFNG [33]. An example of a non-explicit recommendation is ‘choose food low in fat, sugar and salt’. An age-specific food-based dietary guideline that is formulated in quantitative terms [59] will facilitate the interpretation of dietary guidelines more objectively and reduce the various arbitrary choices involved in the construct of an index.

Although some authors suggested that the validity of a diet index should be compared to nutritional biomarkers [30], the use of this ‘gold standard’ is often prohibitive due to its invasive nature and expense. We chose a 4DFR as our reference method as this prospective method is not memory-dependent and hence has less correlated errors with an FFQ compared to a 24-hour diet recall [29]. The main limitation of estimated records is the higher participant burden due to multiple-day recording which may discourage completion [60,61]. Despite careful preparation of the food record as an easy-to-carry booklet and provision of portion aids to facilitate accurate recordings, we acknowledge that misreporting may still occur given the limited motivation and possible poor portion size estimation among adolescents [62,63].

The main limitation of this study was the small sample size (n = 41). The findings of this study must be interpreted with caution as there is a possibility of type one errors due to multiple testing in a small sample. On the other hand, the relatively narrow range of NZDQI-A scores attributed by the low variation in food intakes with the small sample may have restricted the ability to detect true correlations between the two methods for some nutrients. To eliminate learning effects from food recording [29], data from the first administration of the FQ was used to compare with the 4DFR. The reference period of the first FQ (i.e. past seven days or 4 weeks) spanned differently from the 4DFR [32] and may have led to underestimation of the relative validity of NZDQI-A. Nevertheless, the present study yielded some positive findings that suggested that the NZDQI-A is valid as an indicator of diet quality.

The major advantage of the NZDQI-A lies in its simplicity and practicality, as neither nutrient quantification nor food composition data are required for its score derivation. Based on summary questions and frequency questions from the FQ, diet quality was assessed based on intakes of variety and servings of foods recommended for adolescents. Further to extending the good use of dietary information from a brief dietary assessment tool, this index may be applied to assess diet quality in studies of a broad range of adolescent populations, including those where study resources are limited.

## Conclusions

Based on the comparable NZDQI-A scores between the two methods and relatively consistent scores produced when repeated within two weeks, we concluded that the FQ-derived NZDQI-A is relatively valid and reliable for ranking diet quality in adolescents at group level even in a small sample size. Higher NZDQI-A scores were also associated with more favourable nutrient intakes, particularly for fat and iron.

An important implication of this study is the possibility of assessing attributes of diet quality when only limited dietary information is available. The simple NZDQI-A proposed in this study may serve as a tool to rank individuals by their diet quality in surveys. Nevertheless, future research is recommended to examine the ultimate validity of this NZDQI-A in predicting health outcomes.

## Abbreviations

NZ: New Zealand; FFQ: Food frequency questionnaire; 4DFR: Four-day estimated food record; NZDQI-A: New Zealand diet quality index for adolescents; NZAFFQ: New Zealand adolescent food frequency questionnaire; FQ: Food questionnaire; NZFNG: New Zealand food and nutrition guidelines; FRUIT: Fruits; VEGGIE: Vegetables; CEREAL: Bread and cereals; DAIRY: Milk and milk products; MEAT: Meat and alternatives; SFA: Saturated fat; MUFA: Monounsaturated fat; PUFA: Polyunsaturated fat.

## Competing interests

The authors declare that they have no competing interests.

## Authors’ contributions

JEW, KEB, WRP and PMLS were responsible for the study conception and design. JEW, KEB, ASH and PMLS were responsible for data collection. JEW was responsible for the data analyses and drafted the manuscript. All authors provided critical revision of all drafts of the manuscript, and read and approved the final manuscript.

## Pre-publication history

The pre-publication history for this paper can be accessed here:

http://www.biomedcentral.com/1471-2458/13/562/prepub

## Supplementary Material

Additional file 1 Food items included in the New Zealand Diet Quality Index for Adolescents (NZDQI-A) components. Click here for file
