# Supplementary material for: Development and validation of a food-based diet quality index for New Zealand adolescents
Source: BMC Public Health. 2013 Jun 8;13:562. doi: 10.1186/1471-2458-13-562 (PMC3706237; doi:10.1186/1471-2458-13-562)
Supplement: Additional file 1 — Food items included in the New Zealand Diet Quality Index for Adolescents (NZDQI-A) components. [file 1471-2458-13-562-S1.docx]

## Additional file 1 Food items included in the New Zealand Diet Quality Index for Adolescents (NZDQI-A) components.

| Food group (COMPONENT) |  | Food sub groups | Name of items in the NZAFFQ^1^ |
| --- | --- | --- | --- |
| Fruits  (FRUIT) | 1 | Pineapple | Pineapple |
|  | 2 | Apples and pears | Apples, Pears |
|  | 3 | Oranges and mandarins | Oranges or mandarins |
|  | 4 | Bananas | Bananas |
|  | 5 | Kiwifruit | Kiwifruit |
|  | 6 | Other fruits | Peaches or nectarines, Apricots, Plums, Strawberries or berries, Grapes, Melons (including watermelon, rockmelon, honeydew) |
| Vegetables (VEGGIE) | 1 | Cruciferous vegetables | Broccoli or cauliflower, Cabbage or coleslaw, Brussel sprouts |
|  | 2 | Green leafy vegetables | Lettuce or salad green, Mixed vegetables, Watercress or puha, Silverbeet or spinach |
|  | 3 | Marrow-like vegetables | Cucumber, Zucchini or courgette |
|  | 4 | Red or yellow vegetables | Pumpkin, Kumara, Carrots, Capsicums, Tomatoes |
|  | 5 | Potatoes | Hot chips or wedges or French fries, Potatoes (not fried) |
|  | 6 | Other vegetables | Onion or leeks, Mushrooms, Corn, Taro, Peas or green beans, Celery or asparagus. |
| Bread and cereals (CEREAL) | 1 | Breakfast cereals | Breakfast cereals (all kinds) |
|  | 2 | Bread or buns | White bread or roll, Brown or wholemeal bread or roll |
|  | 3 | Rice, pasta or noodles | Rice/ pasta/ noodles |
| Milk and milk products  (DAIRY) | 1 | Milk  (standard and non-standard) | Standard milk (dark blue), Low-fat milk (light blue)/ trim milk (green)/ calci-trim milk (yellow)/ rice milk/ soy milk |
|  | 2 | Flavoured milky drink | Milky or chocolate drink |
|  | 3 | Cheese | Cheese |
|  | 4 | Yoghurt | Yoghurt |
| Meat and alternatives (MEAT ) | 1 | Poultry | Chicken/ turkey/ duck |
|  | 2 | Eggs | Eggs |
|  | 3 | Nuts or seeds | Nuts or seeds |
|  | 4 | Meat alternatives | Tofu/ vegetarian sausages/ falafel |
|  | 5 | Legumes | Baked beans/ chickpeas/ lentils/ kidney beans |
|  | 6 | Red meats | Beef, Lamb or mutton, Pork |
|  | 7 | Fish and seafood | Fish, Other seafood (including mussels, oyster, prawns) |

^1^ 53 food items in the New Zealand Adolescent Food Frequency Questionnaire (NZAFFQ, section 2 of the Food Questionnaire) that were included in the scoring of the NZDQI-A.
